# eSMC: a statistical model to infer admixture events from individual genomics data

**DOI:** 10.1186/s12864-022-09033-2

**Published:** 2022-12-14

**Authors:** Yonghui Wang, Zicheng Zhao, Xinyao Miao, Yinan Wang, Xiaobo Qian, Lingxi Chen, Changfa Wang, Shuaicheng Li

**Affiliations:** 1 Liaocheng Research Institute of Donkey High-Efficiency Breeding and Ecological Feeding, Liaocheng University, 252059 Liaocheng, People’s Republic of China; 2 The Byoryn Technology Co., Ltd, 518122 Shenzhen, People’s Republic of China; 3 Department of Computer Science, City University of Hong Kong, 83 Tat Chee Ave, Kowloon Tong, Hong Kong, People’s Republic of China; 4 School of forensic and medicine, Xi’an Jiaotong University, Xi’an, 710004 Xi’an, Shaanxi, People’s Republic of China; 5 Department of Obstetrics and Gynecology, Peking University Shenzhen Hospital, 518036 Shenzhen, People’s Republic of China; 6 The BGI Education Center, University of Chinese Academy of Sciences, 518083 Shenzhen, People’s Republic of China

**Keywords:** PSMC, Population Admixture, TMRCA, Domestication, Demographic History

## Abstract

**Background:**

Inferring historical population admixture events yield essential insights in understanding a species demographic history. Methods are available to infer admixture events in demographic history with extant genetic data from multiple sources. Due to the deficiency in ancient population genetic data, there lacks a method for admixture inference from a single source. Pairwise Sequentially Markovian Coalescent (PSMC) estimates the historical effective population size from lineage genomes of a single individual, based on the distribution of the most recent common ancestor between the diploid’s alleles. However, PSMC does not infer the admixture event.

**Results:**

Here, we proposed eSMC, an extended PSMC model for admixture inference from a single source. We evaluated our model’s performance on both *in silico* data and real data. We simulated population admixture events at an admixture time range from 5 kya to 100 kya (5 years/generation) with population admix ratio at 1:1, 2:1, 3:1, and 4:1, respectively. The root means the square error is $\pm 7.61$ kya for all experiments. Then we implemented our method to infer the historical admixture events in human, donkey and goat populations. The estimated admixture time for both Han and Tibetan individuals range from 60 kya to 80 kya (25 years/generation), while the estimated admixture time for the domesticated donkeys and the goats ranged from 40 kya to 60 kya (8 years/generation) and 40 kya to 100 kya (6 years/generation), respectively. The estimated admixture times were concordance to the time that domestication occurred in human history.

**Conclusion:**

Our eSMC effectively infers the time of the most recent admixture event in history from a single individual’s genomics data. The source code of eSMC is hosted at https://github.com/zachary-zzc/eSMC.

## Introduction

As a challenge faced by evolutionary biology, the diversity of life history is the foundation of biodiversity [1]. Accelerating the development of sequencing technologies and data analysis methods, individuals and organisms’ genomes have become carriers of evolutionary and ecological events [2]. Reconstructing the demographic histories from genetic data plays an essential role in elucidating the prehistoric events [3, 4]. Population admixture is a ubiquitous feature of demographic history and occurs when isolated populations gather and exchange genetic information [5]. As one reason for anciently diverged alleles, admixture events increase genetic diversity by merging genotypes among populations and masking deleterious mutations [6]. Admixture could increase the fitness of hybrids, reduce gametic isolation, and disrupt local adaptation [7, 8]. As one of the most important types of genetic flow, admixture event span over the history of species evolution. Thus identifying admixture events and admixture time is one of the most essential problem in population study.

Methods and theories have been developed for population history with admixture inference from extant multi-population genetic data [9]. Some ways inferred demographic histories based on allele frequency spectrum (AFS). However, as a computationally challenging method, AFS ignores linkage information [10]. Other non-parametric methods, such as Principal Component Analysis (PCA), could also be used for inferring population structure. However, when the PCA-based method is used in the temporal samples, the sample dates might be ignored, resulting in incomplete plots [11, 12]. Also, all of these method require sequencing data or micro array data of existing populations as input, and estimate the admixture trees or admixture graphs for those input populations. However, more than 1000 species will go extinct, making it almost impossible to observe the historical genetic data [13].

Sequencing several individuals’ whole genome instead of sequencing several loci of many individuals implies a trend in population genetics [14]. Derived from the coalescent theory from the 1980s, inference of the most recent common ancestor (TMRCA) of two or more lineage genomes has been widely used in evolutionary biology [15]. Many approaches have been reported for estimating TMRCA. One way to evaluate TMRCA is to consider multiple genetic neutral markers for multi-population [16]. Another Hidden Markov Model (HMM) based methods could infer TMRCA from the complete chromosome information, such as multiple Sequentially Markovian Coalescent (MSMC) [17] and Pairwise Sequentially Markovian Coalescent (PSMC) [18]. As a computational method, PSMC relies on the distribution of TMRCA between alleles along with a diploid individual genome [19]. PSMCs estimate the historical effective population size from genome-scale data of a single individual [20]. Specifically, the PSMC models use an HMM framework and infer the timing of population divergence and estimate mutation rates and recombination rates [21]. Nevertheless, PSMC does not consider the admixture event in the HMM modeling.

Herein, we developed eSMC, an extended PSMC model, which attaches the admixture time as a free-parameter to model the abrupt increase in effective population size from single individual. eSMC yields the most recent admixture event time and all the results that PSMC should have. To validate the correctness of admixture time inference, we simulated 2000 experiments with the admixture time range from 5 kya to 100 kya (5 generations/year) and the admix ratio at 1:1, 2:1, 3:1, and 4:1, respectively. Our method accurately inferred the admix time with the root mean square error (RMSE) $\pm 7.61 kya$. The model is more accurate at small admix ratio ($RMSE=\pm 5.7 kya$ at ratio 1:1 and $RMSE=\pm 9.75$ at ratio 4:1) and admixture time range from 20 kya to 80 kya. As admix ratio adjacent to 1 represents the large relevant historical effective population size of the admixed subpopulation. The admixture events most recent than 20 kya or later than 100 kya can hardly be identified in the current genome sequence. We also applied our method on four human, five donkey and five goat individuals, respectively. Our model indicated that the admixture events happened at 60 kya to 80 kya for Han and Tibetan individuals (25 years/generation), 40 kya to 60 kya for donkey (8 years/generation) and 60 kya to 100 kya for goats (6 years/generation). The estimated results concordant with the hump start position in PSMC’s historical effective population size curve.

## Implementation

Under the PSMC model, the observed sequence is 100 bp non-overlapping bins along a diploid genome with “.”, “0,” and “1” as values, where “.” representing missing, “0” representing homozygous, and “1” representing heterozygous, respectively.

The method estimates population scaled mutation rate, scaled recombination rate, and piecewise constant effective population size by taking the discrete TMRCA between alleles along the diploid genome as hidden states. The emission probability is $e(1|t) = e^{(-\theta )}$, $e(\theta |t) = 1-e^{(-\theta t)}$ and $e(.|t) = 1$, the transmission probability is

1 $$\begin{aligned} p(t|s) = \left(1 - e^{(-\rho t)} q(t|s)\right) + e^{(-\rho t)} \delta (t - s) \end{aligned}$$

where *t* is the hidden state, $\theta$ is the scaled mutations rate, $\rho$ is the scaled recombination rate, $\delta (.)$ is the Dirac delta function. *q*(*t*|*s*) is a function of the relative effective population size at state t ($\lambda (t) = N_e(t) / N_0$), representing the transmission probability under the condition there being a recombination event.

We consider the admixture events between populations under the following assumptions: 1) the admixture event happens at an instant time, not a duration; 2) the two populations have the same sequence length; 3) the two populations have the same scaled mutation rate $\theta$ and scaled recombination $\rho$; 4) the two populations have the same $N_0$.

Given two populations *P*1 and *P*2 with relevant effective population size $\lambda _a(t)$ and $\lambda _b(t)$. Assuming population *P*2 admixed into population *P*1 at time $t_a$. The relevant effective population size is

2 $$\begin{aligned} \lambda '(t) = \left\{ \begin{array}{lr} \lambda _a(t) &{} if \quad t > t_a \\ \lambda _a(t) + \lambda _b(t) &{} if \quad t \le t_a \end{array} \right. \end{aligned}$$

While the relevant effective population size $\lambda '(t)$ are free parameters for the model, we further look back at the equations of the PSMC model.

**Fig. 1:**
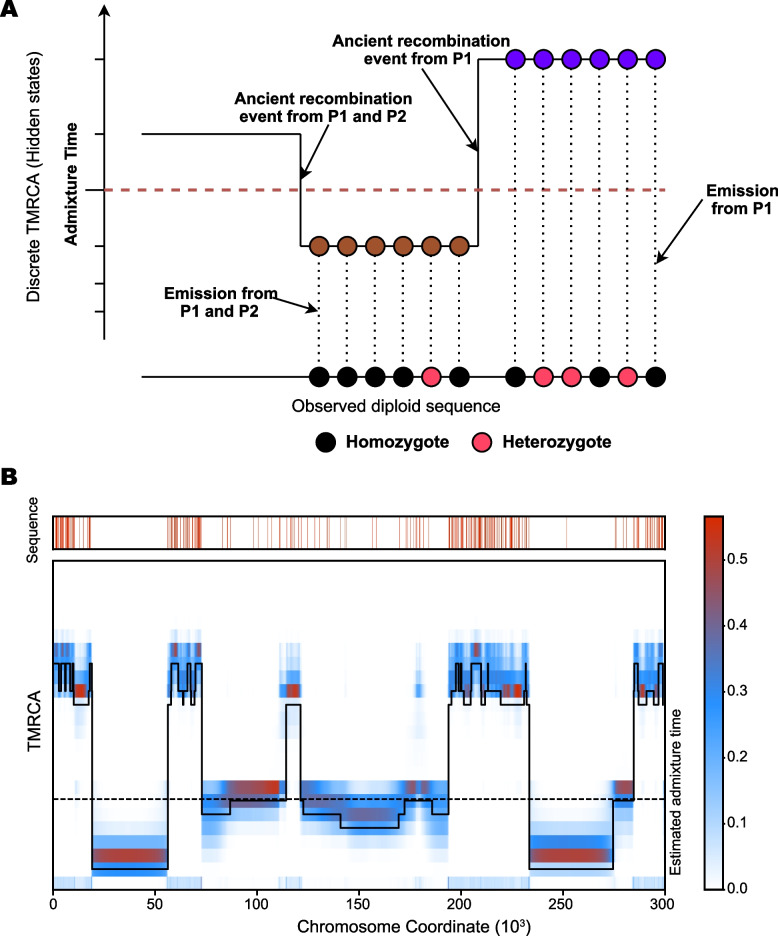
Illustration of the eSMC model. **A** The eSMC using a Pairwise Sequentially Markovian Coalescent model where the observed states are the sequence heterozygosity and the hidden states are the discrete TMRCA. The emission probability and the transition probability is calculated by considering the admixture time $t_a$ of the populations. The brown dots on the on the curve represents TMRCA states after the admixture events, and the blue dots on the curve represents TMRCA states before the admixture event. The P1 and P2 represents the two admixed populations. **B** We applied the eMSC method on a 300 kbp diploid genome simulated by msHOT. The black curve indicate the estimated TMRCA (hidden state) along the genome. The heatmap is the inferred TMRCA probability at each loci with the observed sequence. The estimated admixture time is shown as orange dash line on the heatmap. The red stripes at the simulated sequence indicate the heterozygote positions

The emission probability remains unchanged with the scaled mutation rate and the TMRCA t at loci. Denote *R* as the recombination event at the locus s. $R=1$ stands for a recombination event between *l* and $l+1$, and $R=0$ stands for there is no recombination event between *l* and $l+1$. Denote the conditional transition probability for population *P*1 and *P*2 are $q_a$ and $q_b$. Assuming the hidden state (TMRCA) at *l* is *s*, and the hidden state at $l+1$ is *t*, the conditional transition probability $q'$ has the following conditions (as illustrated in Fig. 1(A)):

1. $t > t_a$ The recombination event happened before the admixture event (the blue dots in Fig. 1(A)). Then the conditional probability will be as same as there is only one population *P*1.
  3 $$\begin{aligned} q'(t|s) = q_a = \frac{1}{\lambda '(t)}\int _{0}^{min\{s,t\}}\frac{1}{s}e^{-\int _{u}^t\frac{dv}{\lambda _a(v)}}du \end{aligned}$$
2. $t \le t_a < s$ The admixture event happened between the two TMRCA time slot *s* and *t* (the vertical curve before brown dots in Fig. 1(A)). Under this circumstance, the recombination event happened at only one population between *P*1 and *P*2.
  4 $$\begin{aligned} q'(t|s)= & {} q_a(1 - q_b) + q_b(1 - q_a) = q_a + q_b - 2 q_a q_b \nonumber \\= & {} \left(\frac{1}{\lambda _a(t)} + \frac{1}{\lambda _b(t)}\right)\int _{0}^{min\{s,t\}}\frac{1}{s}e^{-\int _{u}^t\frac{dv}{\lambda _a(v)}}du \nonumber \\&\quad - 2\int _{0}^{min\{s,t\}}\int _{0}^{min\{s,t\}}\frac{1}{s^2}e^{-\left(\int _{u}^{t}\frac{dv}{\lambda _a(v)} + \int _{m}^{t}\frac{dm}{\lambda _b(v)}\right)}dudm \end{aligned}$$
3. $t \le t_a$ and $s \le t_a$ As the two populations have admixed together. The emission probability will be in the same form with PSMC, where the relevant effective population size will be $\lambda '(t) = \lambda (a) + \lambda (b)$ (the brown dots in Fig. 1(A)).
  5 $$\begin{aligned} q'(t|s) = \frac{1}{\lambda '(t)}\int _{0}^{min\{s,t\}}\frac{1}{s}e^{-\int _{u}^t\frac{dv}{\lambda _a(v)}}du \end{aligned}$$
  The transition matrix, after considering admixture events in the population history, will be
  6 $$\begin{aligned} p^{\prime }(t|s) = (1 - e^{\rho t})q^{\prime } + e^{\rho t}\delta (t - s) \end{aligned}$$

Additional to the PSMC model, we set the admixture time $t_a$ as free parameters. The estimated admixture time is set to 0 at the initial stage of the expectation-maximization (EM). Parameter estimation is conducted between coalescent time intervals in the discrete-state HMM model. Figure 1(B) provide a demo for eSMC. The model captures the increase in effective population size at the admixture event time by the increased frequency of heterozygote markers.

## Results

We verified the effectiveness of eSMC on both simulated and empirical data.

### eSMC can accurately infer admix events in $in\ silico$ experiments

**Fig. 2:**
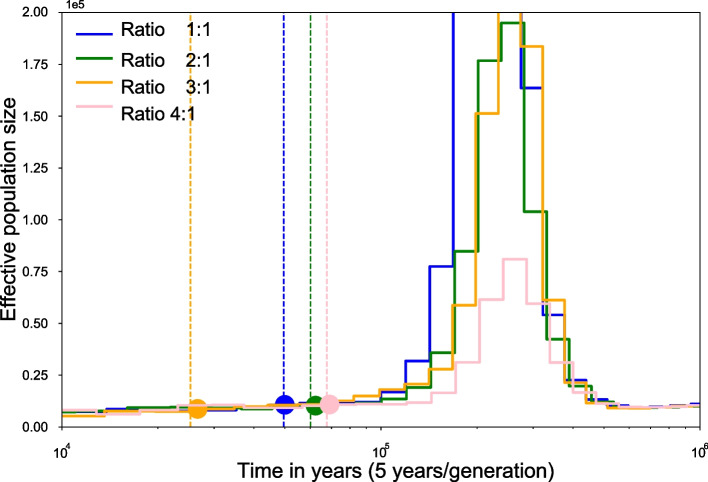
Estimated admixture time and simulated admixture time on PSMC curve. Different colors show the historically effective population sizes of different admix ratios. The dots on the curve are the estimated admixture time by eSMC, and the vertical dash lines are the simulated real admixture time

We subsequently admixed back to a single population for a year ranging from 5 kya to 100 kya. We set the effective population size of the simulated diploid genome to 1*e*5, the years per generation to 5, the mutation rate to $2.5e-8$, the recombination rate to $5e-9$ respectively. The ratio of the effective population size of two diverged sub-populations at the admixture time was set to 1:1, 1:2, 1:3, and 1:4, separately. The estimated historical effective population size, the simulated admixture time, and the estimated admixture time are shown in Fig. 2. The four curves represent the historical effective population size with simulated data at different admixture ratios. The dots represent the estimated admix time, and the vertical dash lines represent the simulated admix time.

**Fig. 3:**
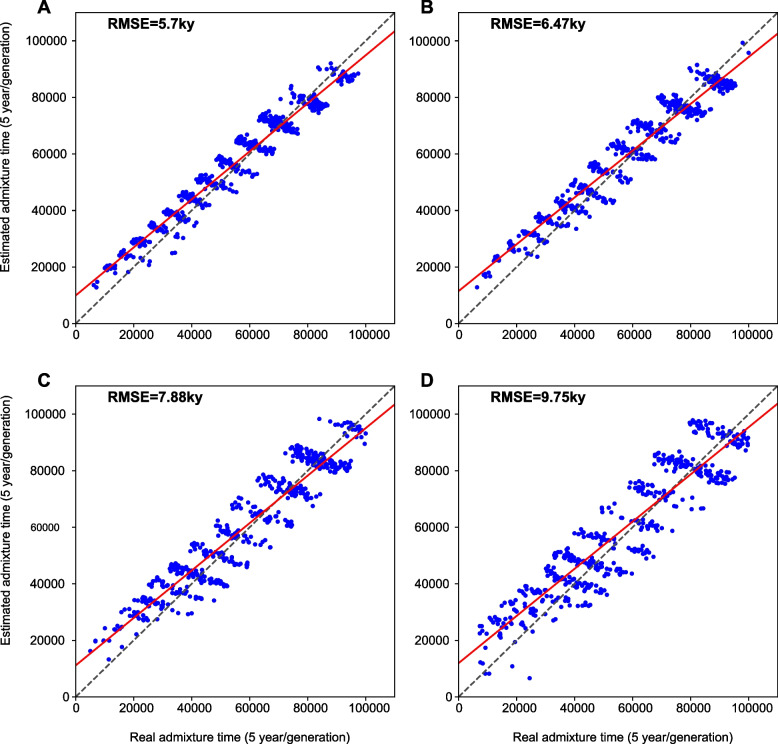
Dot plot for estimated admixture time versus real admixture time in simulated data. Figures **A** to **D** are the dot plots of experiments for admix ratio at 1:1, 2:1, 3:1, and 4:1, respectively. Each dot on the figure represents an experiment, with the x-axis representing the simulated real admixture time and the y-axis representing the estimated admixture time. The black dash diagnosis lines are the datum line. The red lines are the linear regression lines with the simulated data

The results of the experiments are shown in Fig. 3. The x-axis is the simulated admixture time, and the y-axis is the estimated admixture time. RMSE measured the accuracy of our method. The diagonal dash line in black is the data line. The red line is the linear regression line by the experiment time dots. The time dots cluster horizontal steps for all figures as the admixture time was estimated by discrete coalescent time interval. The overall RMSE for all experiments is ${\pm 7.61 ky}$. For admixture ratio 1:1, our method can accurately estimate the admixture time as the dots are closely situated to the datum line. The dots spread dispersed to the datum line when the admixture ratio becomes lower - this concordance with the large RMSE in low admixture ratio. The error is primarily due to the high signal-to-noise ratio, as the admixture event between populations with small effective population size can hardly be captured.

**Fig. 4:**
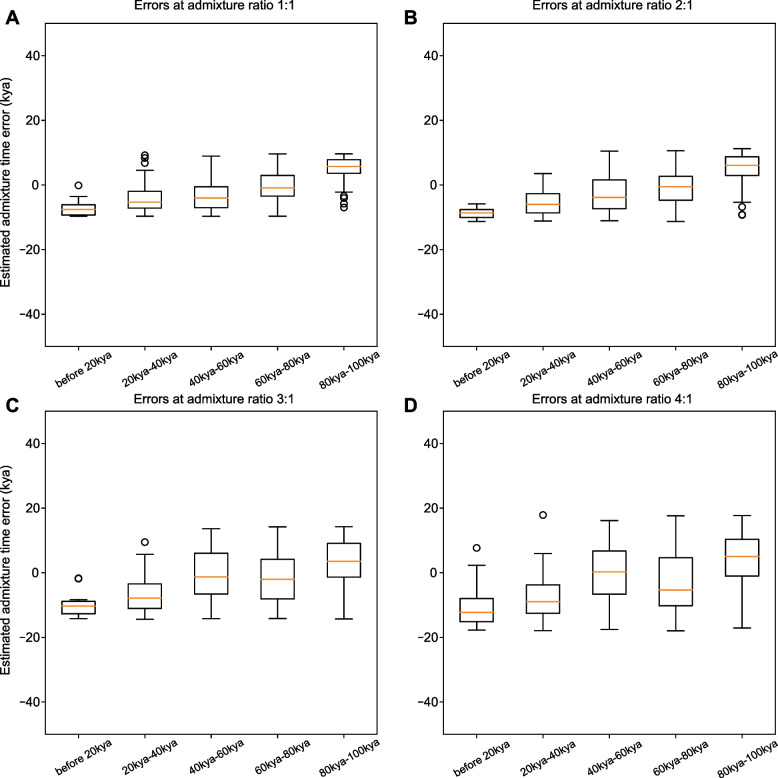
Box plot for experiment errors (the deviation between real admixture time and estimated admixture time) in simulated data. To assess the effectiveness of eSMC at a different time interval, we split the experiments into five-time slots by the simulated admixture time, namely before 20 kya, 20 kya to 40 kya, 40 kya to 60 kya, 60 kya to 80 kya, and 80 kya to 100 kya, respectively. The errors are calculated by the deviation value between the simulated real admixture time and eSMC estimated admixture time

To further explore our method’s effective time interval, we estimated our method’s accuracy at 5-time intervals, namely before 20 kya, 20 kya-40 kya, 40 kya-60 kya, 60 kya-80 kya, and 80 kya-100 kya separately. As shown in Fig. 4, the estimated admixture times are most accurate at 40 kya-80 kya for all admixture ratios. Admixture events in most recent than 20 kya or later than 100 kya can hardly be identified in the current sequence. Our method tends to postpone the admix time at time intervals before 20 kya and prepone the admix time at time intervals 80 kya to 100 kya.

### eSMC’s admix event inference on Han and Tibetan individuals

**Fig. 5:**
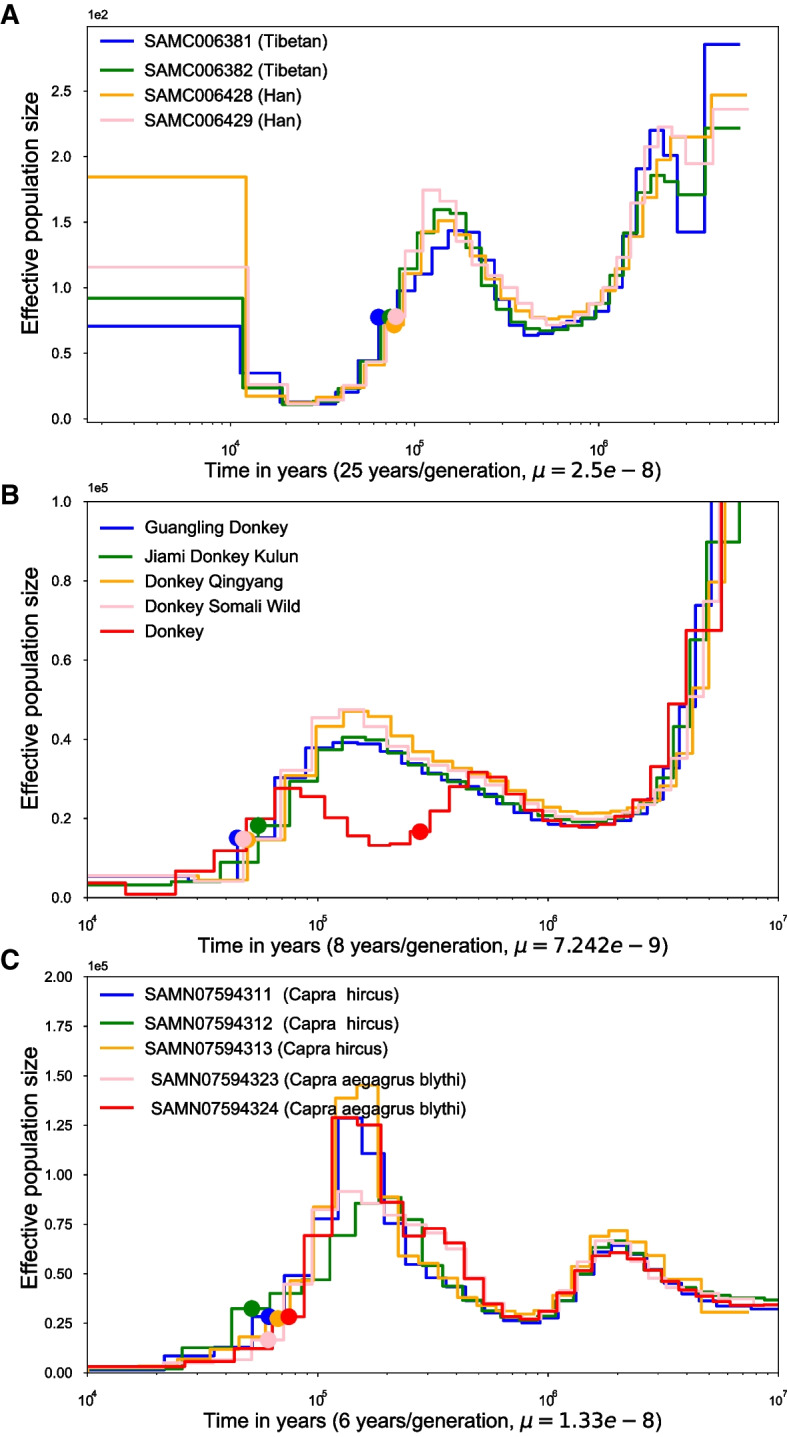
Admix event inference on donkeys and goats. Potential admixture events and admixture times inferred by eSMC. The dots indicated the admixture events on the PSMC effective population size curves. **A** for the Han and Tibetan individuals; **B** for the Somali wild donkey (red) and domesticated donkeys; **C** for the five goat individuals

We downloaded the sequencing Han and Tibetan data from the Genome Sequence Archive (GSA) under accession number PRJCA000246. The selected Tibetan individual IDs were SAMC006381, SAMC006382, and the selected Han individual IDs were SAMC006428, SAMC006429. The downloaded sequencing reads were aligned to GRCh38 by BWA-0.7.17(r1188) with mem command and default parameters. The mutation rate and mutation time were set ${2.5 e-8}$ and 25 years/generation. We performed eSMC to the aligned sequence and inferred the admixture event at 60 kya to 80 kya for both Han populations and Tibetan populations as shown in Fig. 5(A).

The estimated effective population curves were similar to the YRI individuals in PSMC analysis. We observed hump structures in the historical effective population size of the four individuals. The hump structure may generate by population split and admixture events. Our model indicated that the admixture events happened at 60 kya to 80 kya with eight years/generation, concordant with the hump start position in PSMC’s historical effective population size curve.

### eSMC’s admix event inference on Somali wild donkeys and domesticated donkeys

We applied our method to five diploid donkey genomes, as shown in Fig. 5(B). One of them is a Somali wild donkey, while the others are domestic donkeys in Eurasia, namely, Guangling Donkey, Jiami Donkey, Kulun Donkey, and Qingyang Donkey. We downloaded the Somali wild donkey and the four domestic donkeys from the GenBank database under BioProject accession PRJNA431818 and National Genomics Data Center(assession numbers: ERR650540-ERR650547 and ERR650570-ERR650703), respectively. The sequencing data were aligned to a chromosome-level reference genome assembly GCA_016077325.1 [22] by BWA-0.7.17 (r1188) [23] with default parameters. The mutation rate and generation time were set to ${7.242e-9}$ and 8 years/generation according to previous reports [24, 25]. Our model indicated that the admixture events happened at 40 kya to 60 kya with eight years/generation.

Our results consistant with the previous study [24]. The estimated historical effective population size of all the domestic donkeys mixed together. The two ancient donkey populations, *E. africanus somaliensis* and *E. asinus* diverged  0.11 million years ago, and the domestication of the donkeys began at 7 kya to 9 kya [26]. The admixture event of domestic donkeys happened well before the domestication, indicating that the domesticated donkeys may derived from a single source or two sources with a similar biogeography.

### eSMC’s admix event inference on wild and domestic goats

We downloaded the sequencing goat data from the GigaDB dataset (BioProject: PRJNA399234). The selected domestics sample IDs were SAMN07594311, SAMN07594312, SAMN07594313. We also downloaded the sequencing genome of one species of wild goats, namely two samples from *Capra aegagrus blythi*. The sample IDs of *Capra aegagrus blythi* were SAMN07594323 and SAMN07594324. The sequencing data were aligned to a reference genome assembly *Capra hircus* genome V1 by BWA-0.7.17 (r1188) [23] with default parameters [27]. The mutation rate and generation time were set to $1.33e-8$ and 6 years/generation according to previous study [28]. We performed eSMC to the aligned sequence and inferred the admixture event at 40 kya to 100 kya, around 40 kya later than domesticated donkeys as shown in Fig. 5(C).

Both the historical effective population size and the inferred admixture time for domestic goats and wild goats mixed together. This indicate goat breeds are very different compared to most domesticated species. Concordance to the conclusion in previous study that the gene flow among goat populations are probably lacking geographical isolation rather than adherence to pedigree or the use of herd-books [29].

Goats have a larger effective population size compared to donkeys. The historically effective population size of goats has a similar pattern with domesticated donkeys. The indicated admixture time range in or approximate to the Upper Paleolithic or so-called Late Stone Age dates between 12 kya to 50 kya. This period covered half of the Last glacial period with automatic modern human beings emerged. This explains the rapid drop in historical effective population size in goat history and may result in the disappearance and admixture of sub-populations. Domestication occurred afterward with the last glacial period, and human beings started to captive animals.

## Discussion and Conclusion

With the report of the draft genome of Neanderthals, an exploration of human history and origin is constantly unfolding [30]. For non-African populations, about 2% of Neanderthal ancestry was found from modern-day people’s sequencing data. In 2020, a Princeton team developed a method named IBDmix, based on identity by descent (IBD) [31, 32]. IBDmix detected a higher signal of Neanderthal ancestry from African instead of non-African( 30%). Neanderthal DNA in modern humans may have positive and negative effects. Recently, it has been reported that DNA segments inherited from Neanderthals may be closely related to severe COVID-19 infection and hospitalization [33]. Although ancient hominins vanished across history, we still trace their genetic information in modern humans [34]. The potential admixture event in ancient populations may reveal the migration histories and provide hints to archaeology studies.

Large-scale paleogenomics research tends to search for ancient human DNA. Similar research in nonhuman species is also developing [35]. Whole genome-wide data are now easy to obtain due to the continuous development of sequencing technologies [36]. As a sustained transition in human history, the domestication of animals and plants resulted in population admixture and gene flow [22, 37].

Due to intense artificial selection, the process of domestication is usually accompanied by a decrease in genetic diversity and an increase in linkage disequilibrium. Domestication tends to adapt to the “less is more” mode, discarding unnecessary variations based on 2%-4% of human selection [38]. Currently, 28% of domesticated varieties have vanished [39]. Reconstruction of the domestic population structure provides new insights into a biological invasion, farming industry, and global warming [40].

Reconstruction demographic history of the observed population is always used to address anthropological and evolutionary questions [41]. Regarding the classic demographic models, there are three ways to deal with this complex issue [42, 43]. 1) As for multi-population, allele frequency-based methods such as AFS might be a straightforward way. However, this method regards all alleles are dependent. 2) Methods based on IBD or identity-by-state (IBS) could also be a powerful way for inferring demographic models requiring phased data. 3) HMM-based methods provide an effective means of inferring historical demographics in terms of genomic data. It is instrumental when genomic sequences are limited to a few individuals [44]. However, there are challenges in interpreting the output of PSMC and MSMC, as the underlying models for testing hypotheses usually require more available data [45].

Our method takes the genomic data of one individual as the input to infer the most recent admixture event in its population history. We verified our method on simulated data with different admixture ratios, and applied our methed on real data, including human individuals, wild and domesticated donkeys as well as goats to infer their historical population demographics and further insights into their domesticating history.

Our model can hardly estimate the admixture events in most recent than 20 kya or later than 100 kya (for 5 years per generation). As expected, the height of the hump in the historical effective population size generated by admixture events steps down to the effective population size of the admixed sub-population. Thus, our method eSMC can hardly handle admixture events for sub-populations with small effective population size (large admix ratio). Moreover, sequencing data from multiple individuals can provide more reliable and accurate information in admixture event estimation. We will modify our model to make it feasible for multiple individuals in the sample population.

## Availability and requirements

**Project name:** eSMC

**Project home page:** https://github.com/zachary-zzc/eSMC

**Operating system(s):** Platform independent

**Programming language:** Shell script, Python script, C++

**License:** see web page

**Any restrictions to use by non-academics:** license needed.

## Data Availability

The genome sequencing data processed in this study are downloaded from public database. We downloaded the genome sequencing data of human individuals from Tibetan and Chinese Han population are from Genome Sequence Archive (GSA) under accession number PRJCA000246. The domestic donkeys and wild somali donkey data were collected at GenBank database under BioProject accession PRJNA431818 and National Genomics Data Center(assession numbers: ERR650540-ERR650547 and ERR650570-ERR650703). The goats individuals are available at GigaDB dataset under BioProject accession number PRJNA399234. The source code and scripts are available in the [eSMC] repository, https://github.com/zachary-zzc/eSMC.

## Abbreviations

PSMC: Pairwise Sequentially Markovian Coalescent
eSMC: extended Pairwise Sequentially Markovian Coalescent
AFS: allele frequency spectrum
PCA: Principal Component Analysis
TMRCA: the most recent common ancestor
MSMC: multiple Sequentially Markovian Coalescent
HMM: Hidden Markov Model
